# Improved Reconstruction of Radio Holographic Signal for Forward Scatter Radar Imaging

**DOI:** 10.3390/s16050651

**Published:** 2016-05-07

**Authors:** Cheng Hu, Changjiang Liu, Rui Wang, Tao Zeng

**Affiliations:** 1 Beijing Key Laboratory of Embedded Real-time Information Processing Technology, School of Information and Electronics, Beijing Institute of Technology, Beijing 100081, China; cchchb@163.com (C.H.); liuchangjiang@bit.edu.cn (C.L.); zengtao@bit.edu.cn (T.Z.); 2 Department of Electronic Engineering, Tsinghua University, Beijing 100084, China

**Keywords:** forward scatter radar, radio holographic signal, Hilbert transform, radar imaging

## Abstract

Forward scatter radar (FSR), as a specially configured bistatic radar, is provided with the capabilities of target recognition and classification by the Shadow Inverse Synthetic Aperture Radar (SISAR) imaging technology. This paper mainly discusses the reconstruction of radio holographic signal (RHS), which is an important procedure in the signal processing of FSR SISAR imaging. Based on the analysis of signal characteristics, the method for RHS reconstruction is improved in two parts: the segmental Hilbert transformation and the reconstruction of mainlobe RHS. In addition, a quantitative analysis of the method’s applicability is presented by distinguishing between the near field and far field in forward scattering. Simulation results validated the method’s advantages in improving the accuracy of RHS reconstruction and imaging.

## 1. Introduction

**Figure 1:**
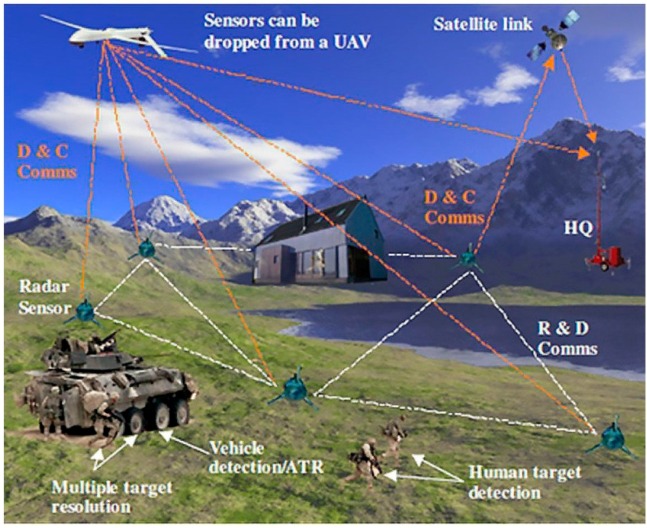
Illustration of netted forward scattering detection. ATR: Automatic Target Recognition; UAV: Unmanned Aerial Vehicle; D & C Comms: Data and Control Communications; R & D Comms: Radar and Data Communications [11].

Forward scatter radar (FSR) is usually classified as bistatic radar for its separated transmitter and receiver. Targets detected by FSR have bistatic angles close to 180° [1]. In other words, they are in the forward scattering (FS) region [1]. Radar cross sections (RCS) of targets in the FS region are larger than those of targets in the monostatic configuration and, furthermore, are independent of the targets’ surface material. These special characteristics give FSR the potential to detect stealth targets [2]. As one of the new radar systems, FSR has become a hot research point in the 21st century. Many published studies have applied FSR to air target detection [3], maritime surveillance [4], and ground target recognition and classification [5,6,7,8]. Aside from ground-based FSR, navigation satellites have also been used for bistatic remote sensing in the FS region, such as remote sensing imaging [9] and moving target detection [10]. Using netted FSR sensors connected by wireless communication, the situational awareness of certain districts can be achieved. Furthermore, the surveillance of a wide range of airspace can be also realized by the use of FSR sensors cooperating with satellites, as shown in Figure 1 [11].

To develop the ability of target recognition in FSR, the concept of shadow inverse synthetic aperture radar (SISAR) was first proposed by Chapurskiy *et al.* [12,13] in the 1980s and has attracted more attention since the IEEE radar conference held in 2000 [14]. According to the SISAR theory, the FS echo of a target in the Fresnel zone can be expressed as the Fresnel transform of the target‘s complex profile function (CPF) [13], from which the target’s shadow profile information can be extracted. Successively, the clutter suppression problem was investigated in [15]. A motion compensation method was proposed in [16,17]. In recent years, the signal modeling and imaging algorithms under circumstances of big diffraction angle, multipath intervene, and non-perpendicular crossing targets were improved in [18,19,20].

Different from the traditional point target imaging technique for inverse synthetic aperture radar (ISAR), SISAR imaging is more like the holographic imaging in optical diffraction theory. As a result, the complex echo signal used for SISAR imaging is called radio holographic signal (RHS) [12,13,14]. The acquisition of RHS is the prerequisite for SISAR imaging. In FSR signal processing, synchronous processing is needed to obtain the RHS from the carrier. A direct way to achieve synchronization is connecting the transmitter and the receiver by cables, which is only used in situations of very short baselines for its high cost and loss [21]. Another way is to use synchronous clocks with considerably high frequency instabilities, which is also impractical. As can be concluded, the signal at the input of the receiving antenna in FSR can be represented as a sum of the strong direct signal and the weak target return. Thereby, a substitutive method has been widely used to obtain one quadrature component of RHS by means of an amplitude detector [22]. However, only one quadrature component of RHS is not enough for SISAR imaging, making most of the earlier research on SISAR based on simulated data. It can be seen that the reconstruction of RHS is a major problem in FSR signal processing. 

In [23], a Hilbert transform was used to reconstruct a complex FS signal for detection and parameter estimation. Theoretically, this is only effective on signals with short durations and nonzero Doppler. A method for RHS reconstruction based on segmented Hilbert transform and second-order fitting was proposed in [24]. However, it is found that this method contains some defects. Firstly, the segmented Hilbert transform cannot ensure the zero mean property of reconstructed signals, which means that local means of reconstructed RHS should be always zeroes. Secondly, as the initial phase of RHS is uncertain, using quadratic polynomial fitting to reconstruct the other quadrature component of RHS is not applicable to all cases.

To address the above problems, this paper proposes an improved method for RHS reconstruction. Firstly, a different way of segmented Hilbert transform is employed to ensure the zero mean property of reconstructed RHS. Then, extremums of the quadrature component of RHS that has been acquired by envelope detection are used to estimate the amplitude envelope of RHS by high-order fitting. Finally, the quadrature component of RHS acquired by envelope detection, the other quadrature component of RHS acquired by segmented Hilbert transform, and the amplitude envelope acquired by extremum fitting are fused to reconstruct the RHS. The proposed method overcomes many problems of the traditional method and has much greater stability.

The remainder of the paper is organized as follows: In Section 2, the RHS model for SISAR imaging is given and its amplitude/phase characteristics are analyzed in detail. In the next section, the improved RHS reconstruction is discussed from two aspects: segmented Hilbert transform and the reconstruction of mainlobe signal. Meanwhile, the performance of the given method is also analyzed. In Section 4, simulated data are used for the validation of the given method. The paper concludes with a summary of the method’s effects and possible applications.

## 2. RHS Model and Characteristics

### 2.1. RHS Model for SISAR

**Figure 2:**
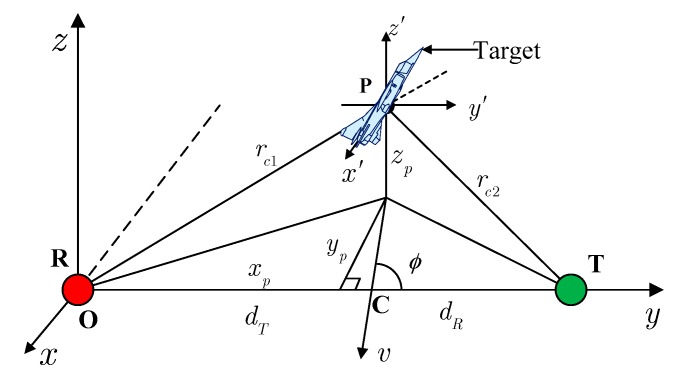
Typical forward scatter radar (FSR) topology.

The topology of a typical FSR system is shown in Figure 2.

The receiver locates in the origin of a Cartesian coordinate system (x,y,z), and the transmitter’s position in this coordinate system is (L,0,0), in which L is the length of the baseline. The local coordinate system $\left(x^{\prime },y^{\prime },z^{\prime }\right)$, the origin of which $\left(x_{p},y_{p},z_{p}\right)$ is the target’s center **P**, is parallel to the global coordinate system (x,y,z). The distances from the target’s center to the transmitter and receiver are represented by $r_{c1}$ and $r_{c2}$, respectively. The target is assumed to move linearly in a plane parallel to the x−y plane with a velocity of v. The angle between the direction of the velocity and the baseline, named track angle, is expressed as ϕ. The projection of the target’s center to the x−y plane when crossing baseline, expressed as **C**, has bistatic distances $d_{T}$ and $d_{R}$. According to the electromagnetic theory, the target’s FS field can be written in the form:

(1) $$E=A\cdot \frac{1}{r_{c1}r_{c2}}\cdot \exp\left[jk\left(r_{c1}+r_{c2}\right)\right]\cdot {\dot{\sigma }}_{s}$$

where A is a complex constant determined by system parameters, k=2π/λ is the wave number, λ is the wavelength of harmonic wave, ${\dot{\sigma }}_{s}$ is the target’s complex scatter coefficient, the amplitude of which is equivalent to the target’s RCS and the phase of which is determined by the target’s scatter character. Assuming that the target crosses the baseline at time t=0 and the diffraction angles are small, the time-domain RHS model can be expressed as [13]:

(2) $$E\left(t\right)=Q\cdot \int_{-l/2}^{l/2}H\left(x^{\prime }\right)\exp\left[j\frac{\gamma }{2}{\left(\frac{x^{\prime }}{v}+t\right)}^{2}\right]dx^{\prime }$$

where l is the maximum shadow length existing at $\phi =\pi /2,z_{p}=0$. The parameter Q describes the phase delay and the amplitude attenuation caused by signal propagation, given by:

(3) $$Q=\frac{A\sin\phi }{j\lambda r_{c1}r_{c2}}\exp\left(j2\pi \frac{L}{\lambda }+j\Omega^{2}z_{p}^{2}\right)$$

In Equation (2), the meaning of the value $\gamma =2\Omega^{2}v^{2}\sin^{2}\phi$ is the rate of Doppler frequency variation of point **P**. The parameter $\Omega =\sqrt{\pi \left(1/d_{T}+1/d_{R}\right)/\lambda }$ can be considered as time-independent. $H\left(x^{\prime }\right)$ stands for the target’s CPF, the amplitude and phase of which contain information of the height difference and median line of the target’s profile. For specific extraction of the target profile, one can refer to [17].

The basic SISAR algorithm under the small diffraction angle assumption is given based on Fresnel diffraction integral. Since Equation (2), has the form of Fresnel transform, by taking the inverse Fresnel transform of E(t) we can obtain the target’s CPF as follows:

(4) $$H^{\prime }(x^{\prime })=\frac{\gamma }{2\pi vQ}e^{-j\frac{\gamma {x^{\prime }}^{2}}{2v^{2}}}\int_{-T_{s}/2}^{+T_{s}/2}e^{-j\gamma \left(t^{2}/2+x^{\prime }t/v\right)}E(t)dt$$

where $T_{s}$ is the synthesizing interval.

### 2.2. RHS Characteristics

To achieve a better performance in RHS reconstruction, thorough analysis and understanding of the characteristics of RHS are quite necessary. The RHS characteristics contain mainly two aspects, which are envelope characteristic and phase characteristic. The phase characteristic is determined by the variation of bistatic distance with time and the complex scatter coefficient, while the envelope characteristic mainly depends on the target’s FS radar cross section (RCS) [3]. Actually, it is the characteristics of the target’s complex scatter coefficient that are of more concern to us and determine the performance of RHS reconstruction. Published experimental results show that, in far-field scattering, the RHS approximates the chirp signal well. While in near-field scattering, as the shadow radiation effect gets stronger, the complex scatter coefficient will cause obvious modulations of the signal’s amplitude and phase [3,24].

**Figure 3:**
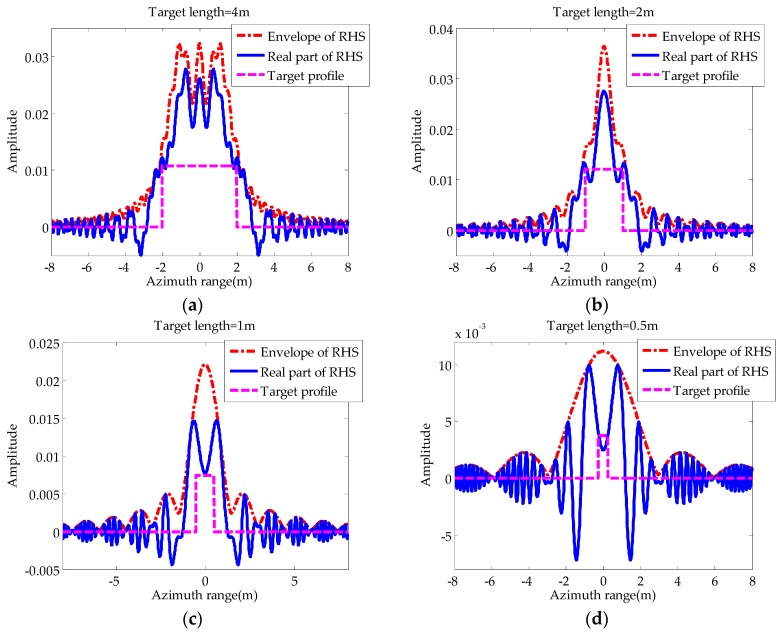
Radio holographic signals (RHSs) of targets with different lengths: (**a**) Target length = 4 m; (**b**) target length = 2 m; (**c**) target length = 1 m; (**d**) target length = 0.5 m.

Next, to make a simplified and quantitative analysis of the differences between the scattering in near-field and far-field, we will simulate the RHS of a rectangle target for illustration. Chapurskiy [10] pointed out that, in the far zone, the RHS amplitude of a rectangular plate with projection length lsinϕ and zero altitude could be approximated by the following form:

(5) $$\left|E\left(t\right)\right|=E_{0}\left|\sin c\left(\gamma lt/2\pi v\right)\right|$$

where $E_{0}=S/\left(d_{T}d_{R}\right)$, S is the shadow area. Equation (5) shows that the envelope of RHS has the form of sinc function. To study the characteristics of RHS, we simulated the RHSs of rectangles with different lengths as shown in Figure 3, and the corresponding simulation parameters are listed in Table 1.

In Figure 3, the RHS envelope is represented by dash-dotted line, the RHS real part is represented by solid line, and the rectangle model, the height of which is normalized using one third of the envelope maximum, is represented by broken line. The abscissa is the moving distance at azimuth.

It is shown that there is considerable diversity in the RHS envelopes of targets with different lengths. For a target of shorter size (e.g., 0.5 m), the far-field diffraction will be dominant, in which case the shape of the envelope resembles a sinc function and the mainlobe of scattered energy is relatively wider. As the length of the target increases, the ratio of the 3 dB width of signal energy to the target length becomes higher, which is an indication of shadow blocking. Besides, more sidelobes appear and their maximum points are higher. When the target length is 4 m, the RHS envelope has sharp fluctuations and completely deviates from the form of the sinc function, an indication of near-field diffraction.

Further, the characteristics of RHS phase will be analyzed. From Equation (1), the RHS phase, expressed by φ(t), is determined by the Doppler phase $k\left(r_{c1}+r_{c2}\right)$, the scatter coefficient phase $\varphi_{\sigma }$, and the initial phase $\varphi_{0}$:

(6) $$\varphi \left(t\right)=k\left(r_{c1}+r_{c2}\right)+\varphi_{\sigma }+\varphi_{0}$$

As we know, when the target size is small enough to be approximated as a point target (*i.e.*, a constant $\varphi_{\sigma }$) , the RHS phase is mainly affected by the Doppler phase, which can be approximated as a quadratic of time. In other words, the signal is linear frequency modulated. However, when the target is large in size and the variation of $\varphi_{\sigma }$ should not be ignored, there will be phase transitions at the minima of the RHS envelope. As a result, the zero-mean property of the quadrature component of RHS within a short time will be interfered. From Figure 3 we can see that the RHS is far from a chirp signal if the frequency of phase transitions is comparable to the Doppler. Additionally, the sign of the quadrature component of RHS remains constant for a long time when a large target is crossing the baseline, indicating that the shadow effect is at near field.

Based on the above analysis, the differences in RHS characteristics between far-field diffraction and near-field diffraction are explicitly shown. It can be concluded that the RHS of far-field diffraction has a smooth envelope and a chirp-like quadrature component, while the RHS of near-field diffraction has a fluctuant envelope and manifests an obvious effect of shadow blocking. Generally speaking, if the scatter mechanism is considered as near-field diffraction, it is very difficult to extract useful information for parameter estimation, owing to the unknown scatter phase [3], and the RHS cannot be accurately reconstructed [15].

## 3. Method

The traditional RHS reconstruction method consists of three procedures: envelope detection, segmented Hilbert transform, and amplitude compensation. Envelope detection can eliminate the influence of direct signal and obtain one quadrature component of RHS represented by I(t) [22,24]. Firstly, we will give the detailed derivation of the model of the signal at the output of the envelope detection. Disregarding the interferences and noise, the complex signal at the input of the amplitude detector, $u_{R}\left(t\right)$, can be presented as a sum of the direct signal and the target return:

(7) $$u_{R}\left(t\right)=U_{dir}\exp\left[j\left(\omega_{0}t+\varphi_{x}\right)\right]+U_{tg}\left(t\right)\exp\left[j\left(\omega_{0}t+\varphi_{D}+\varphi_{\sigma }+\varphi_{0}\right)\right]$$

where $\omega_{0}=2\pi f_{0}$, $f_{0}$ is the carrier frequency, $U_{dir}$ is the amplitude of the direct signal and $U_{tg}$ is the amplitude of the target return. $\varphi_{D}$, $\varphi_{x}$, $\varphi_{0}$ and $\varphi_{\sigma }$ denote the Doppler phase, the initial phase of direct signal, the initial phase of target return, and the scatter phase, respectively. Applying the condition that $U_{dir}\gg U_{tg}$, which is true for a far-field forward scattering [22], we can calculate the amplitude of $u_{R}\left(t\right)$ as :

(8) $$\begin{array}{l} U\left(t\right)=\left|u_{R}\left(t\right)\right|=\sqrt{U_{dir}^{2}+U_{tg}^{2}\left(t\right)+2U_{dir}U_{tg}\left(t\right)\cos\left(\varphi_{D}+\varphi_{\sigma }+\varphi_{x}-\varphi_{0}\right)}\\ \;\approx U_{dir}+U_{tg}\left(t\right)\cos\left(\varphi_{D}+\varphi_{\sigma }+\varphi_{x}-\varphi_{0}\right) \end{array}$$

where the second-order Taylor expansion was used for simplification. Actually, U(t) is the output of the amplitude detector. Since the initial phase $\varphi_{x}-\varphi_{0}$ is difficult to know, we can consider the expression $U_{tg}\left(t\right)\cos\left(\varphi_{D}+\varphi_{\sigma }+\varphi_{x}-\varphi_{0}\right)$ as either quadrature component of RHS. Though the output of the amplitude detector is not exactly equivalent to the quadrature component of RHS, the error introduced by approximation can be ignored for a target in the far-field of forward scattering.

Then, the segmented Hilbert transform can correctly reconstruct most of the other quadrature component of RHS, represented by Q(t). It should be mentioned that the denotation I(t) and Q(t) are not exactly the output of quadrature demodulation and should not be confused. Finally, the amplitude compensation is applied to reconstruct the mainlobe RHS to acquire a whole RHS. Here the mainlobe refers to the central part of RHS that occupies most of the signal power. For far-field scattering, the mainlobe RHS refers to the part of RHS between the first nulls of the RHS envelope. The novel method proposed in this article mainly improves the traditional method in two parts: segmented Hilbert transform and mainlobe RHS reconstruction.

### 3.1. Improved Segmented Hilbert Transform

Hilbert transform is the basic technique to reconstruct a complex signal from its real part. However, the Hilbert transform of the real part of every non-stationary signal is not necessarily its analytic signal. Actually, Bedrosian’s theorem can be applied to explain the prerequisite for the Hilbert transform as follows [25].

If a real amplitude-modulated and frequency-modulated signal s(t)=a(t)cosψ(t) satisfies the conditions that the spectrum of its envelope is inside the region $\left[-f_{0},f_{0}\right]$ while the spectrum of cosψ(t) is outside the above region:

(9) $$\begin{array}{ccc} A\left(f\right)=F\left\{a\left(t\right)\right\}=0 & & f\notin \left[-f_{0},f_{0}\right]\\ B\left(f\right)=F\left\{\cos\psi \left(t\right)\right\}=0 & & f\in \left[-f_{0},f_{0}\right] \end{array}$$

the analytical signal of s(t) has the form presented in Equation (10) as:

(10) $$z\left(t\right)=a\left(t\right)\cos\psi \left(t\right)+jH\left\{a\left(t\right)\cos\psi \left(t\right)\right\}=a\left(t\right)e^{j\psi \left(t\right)}$$

where H{⋅} denotes the Hilbert transform.

**Figure 4:**
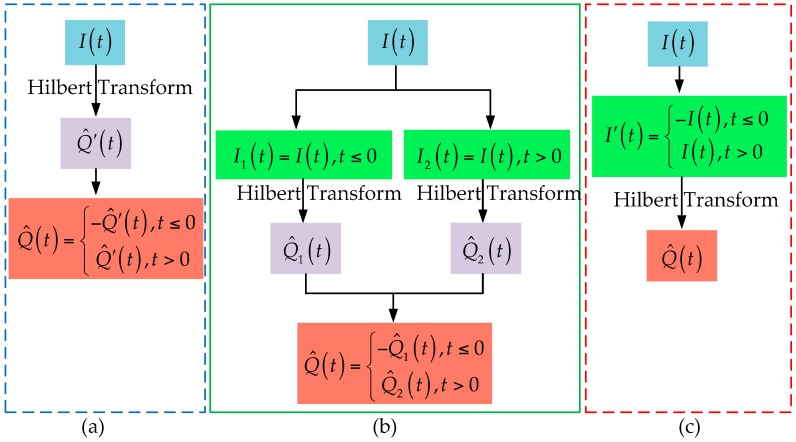
Three ways of segmented Hilbert transform: (**a**) Approach 1; (**b**) Approach 2; (**c**) Approach 3.

Since the Doppler of the FS signal is zero when the target is crossing baseline, the above condition is obviously unsatisfied. If directly taking the Hilbert transform of I(t), the obtained signal will not be the RHS. To solve this problem, the segmented Hilbert transform is proposed to reconstruct Q(t) in [24] and two different implementations are discussed. In the first implementation, the Hilbert transform is taken before the segmentation processing and sign change processing. While in the second implementation, the Hilbert transform is taken between the segmentation processing and sign change processing. In fact, there is a third way of segmented Hilbert transform which is to take the Hilbert transform after segmentation processing and sign change processing. The corresponding illustrations are shown in Figure 4. For the purpose of convenient expression, we will refer to the above three ways of Hilbert transform as Approach 1, Approach 2, and Approach 3, respectively, in the following paragraphs.

**Figure 5:**
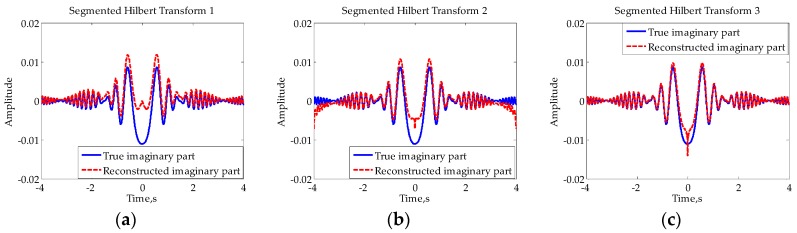
Comparisons of the results of different segmented Hilbert transforms: (**a**) Approach 1; (**b**) Approach 2; (**c**) Approach 3.

The essential difference between the three methods is the relative position of the Hilbert transform to segmentation processing and sign change processing: before, between, and after. For comparison, Figure 5 shows the reconstructed imaginary parts of the signal in Figure 2d using the above three methods, in which the broken lines denote the reconstructed imaginary parts while the solid lines denote the true imaginary parts.

Overall, all of the reconstructed RHSs have obvious distortion at times when Doppler is close to zero. There are also electric mean shifts in the first two cases, which means that local means of signal deviate from zeroes. One has obvious electric mean shift at low frequency while the other has obvious electric mean shift at high frequency. However, the RHS reconstructed using the novel method (Approach 3) represents no obvious electric mean shift. The reason for this phenomenon is that the frequency of the constructed input of the Hilbert transform is not zero at the crossing time, which meets the conditions for the Hilbert transform to a certain extent. In [24], only the electric mean shifts at low frequency are considered and Approach 2 is considered to be a preferable choice. Obviously, Approach 3 should be the best choice if all the three approaches are considered.

### 3.2. Mainlobe RHS Reconstruction

**Figure 6:**
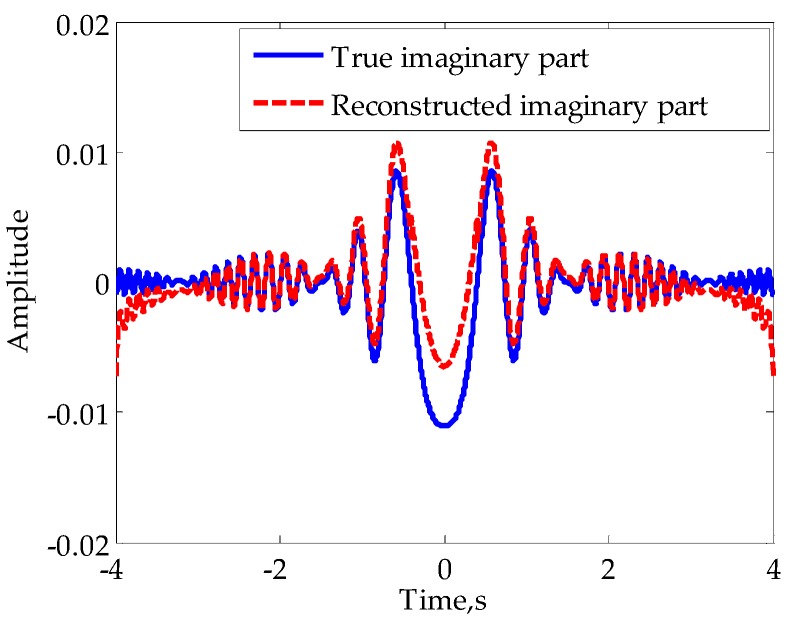
Reconstructed mainlobe RHS using the traditional method.

The traditional method for the reconstruction of mainlobe RHS is based on quadratic polynomial fitting. With the assumption that the initial phase of RHS is zero and I(t) is the real part of RHS, Q(t) can be approximated as $\sin t^{2}$ when t is close to zero. However, according to the measured data of FS signal appearing in published papers [3,4,5,6,12], I(t) is not necessarily the real part of RHS with zero initial phase. On the other hand, the expression of Equation (3) also indicates that the initial phases are different for different system parameters. Therefore, if the quadratic function fitting is used to reconstruct Q(t) when the assumption is not satisfied, a distortion is inevitable. We reconstruct the mainlobe RHS of the signal in Figure 5b using the method in [24] and show the result in Figure 6. It can be indicated that there is further room for the improvement of the reconstruction of mainlobe RHS.

Actually, we can introduce some additional prior information to assist with the reconstruction of Q(t). Regardless of the scattering phase in the case of far-field diffraction, the RHS can be expressed as:

(11) $$E\left(t\right)\approx A\left(t\right)\exp\left(j\gamma t^{2}/2+j\varphi_{0}\right)$$

where A(t) is the envelope and $\varphi_{0}$ is the initial phase. Thus, the I(t) obtained by envelope detection is:

(12) I(t)=Re{E(t)}=A(t)cos(γt2/2+φ0)

Considering the phase, there are two unknown parameters in I(t), which are the Doppler variation rate γ and the initial phase $\varphi_{0}$. If the envelope A(t) is known, we can obtain the estimates of γ and $\varphi_{0}$ by matching I(t) with constructed matching signals in the least square sense, and hence restore Q(t). In fact, however, as the envelope A(t) is unknown, parameter estimation can be only achieved under the assumption that A(t) is constant [4]. Simulation proves that the estimated γ and $\varphi_{0}$ under this assumption have certain deviations from the true values and the reconstructed RHS’s are not accurate.

**Figure 7:**
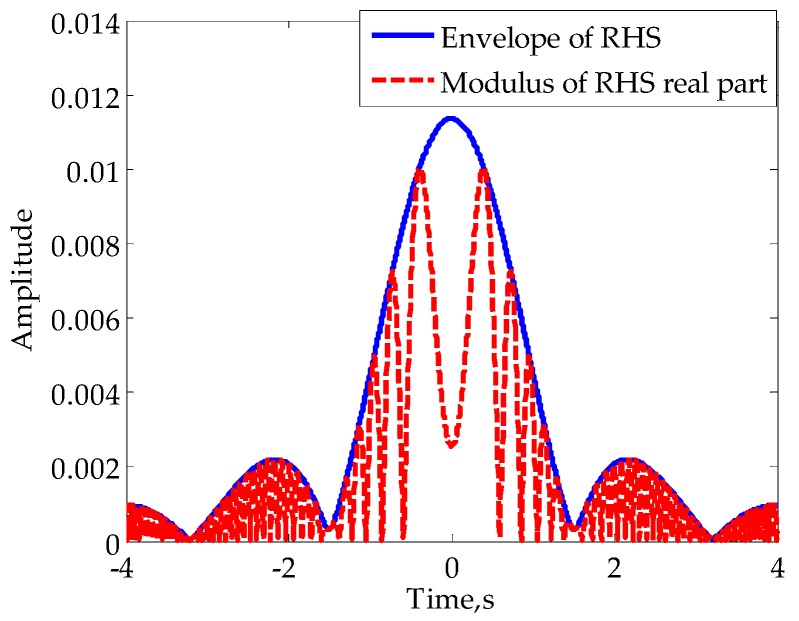
Signal envelope and modulus of the real part.

According to the above analysis, the estimation of the A(t) in mainlobe is the key to RHS reconstruction. From Equation (12) it can be seen that when $\gamma t^{2}/2+\varphi_{0}=n\pi$,n=⋯−2,−1,0,1,2,⋯, we have |I(t)|=A(t). That is to say, the modulus of I(t) is equal to the amplitude of RHS in some specific moments. In particular, when the target is in the far-field, these moments are very close to moments when I(t) reaches its extremums. Therefore, if we can obtain *M* extremums of I(t) in mainlobe (zero moment excluded), expressed as$I\left(t_{m}\right)$andm=1,2,⋯,M, it is appropriate to consider $\left|I\left(t_{m}\right)\right|$ as *M* non-uniform samples of A(t). For illustration, the envelope (denoted by solid line) and modulus of the real part (denoted by broken line) of the signal in Figure 3d are shown in Figure 7.

**Figure 8:**
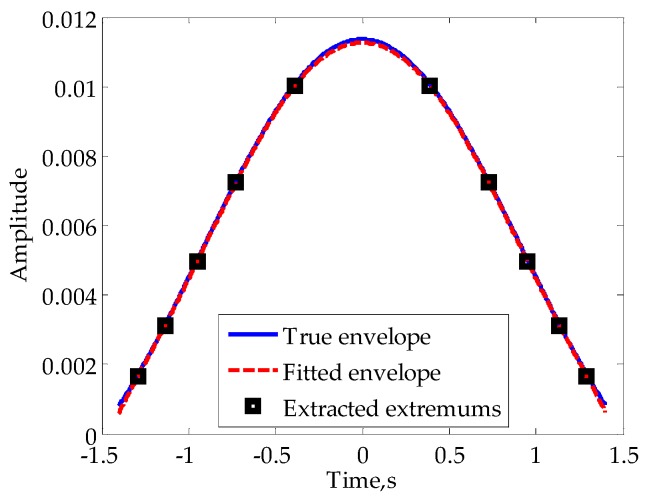
Fitting of the envelope in mainlobe.

For far-field diffraction, the mainlobe envelope is usually smooth. Thus, we can consider the use of high order polynomial fitting to restore the mainlobe envelope in cases of large *M*. For the signal in Figure 7, we extract the extremums in mainlobe, 10 points altogether, to process a fifth-order polynomial fitting. The corresponding result is shown in Figure 8, from which we can see that there are minor differences between the fitting envelope and its true value.

**Figure 9:**
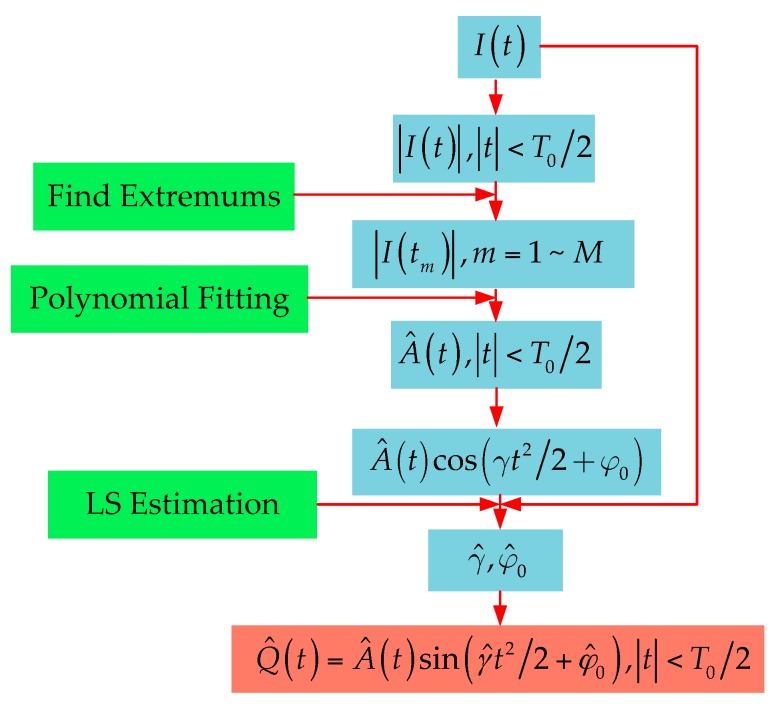
Flowchart for the reconstruction of RHS in mainlobe.

Based on the above description, we present the following method of mainlobe RHS reconstruction, the specific flowchart of which is shown in Figure 9.

**Step 1**: Ascertain the range of the mainlobe $\left[-T_{0}/2,T_{0}/2\right]$ according to the variation of signal amplitude.

**Step 2**: Pick the extremums in the mainlobes $I\left(t_{m}\right)$.

**Step 3**: Use $I\left(t_{m}\right)$ to fit the mainlobe envelope as $\hat{A}\left(t\right)$.

**Step 4**: Estimate the signal parameters γ and $\varphi_{0}$ in the sense of least square error:

(13) $$\left(\hat{\gamma },{\hat{\varphi }}_{0}\right)=\arg\underset{\gamma ,\varphi_{0}}{\min}\left\{\frac{1}{N}\sum_{n=0}^{N-1}{\left[\hat{A}\left(t_{n}\right)\cos\left(\varphi_{0}+\gamma t_{n}^{2}/2\right)-I\left(t_{n}\right)\right]}^{2}\right\},t_{n}\in \left[-T_{0}/2,T_{0}/2\right]$$

where $t_{n}$ represents for the time samples in the mainlobe.

**Step 5**: Substitute $\hat{\gamma },{\hat{\varphi }}_{0}$ into Equation (11) to obtain the estimate of the imaginary part of RHS:

(14) $$\hat{Q}\left(t\right)=\hat{A}\left(t\right)\sin\left(\hat{\gamma }t^{2}/2+{\hat{\varphi }}_{0}\right),\left|t\right|<\frac{T_{0}}{2}$$

**Figure 10:**
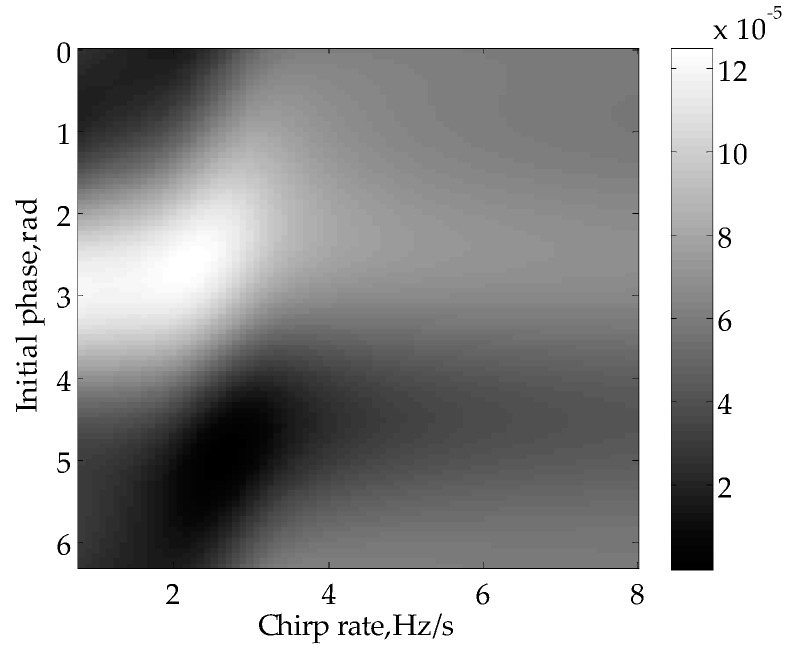
Mean square error distribution in parameter estimation.

The two-dimensional distribution of mean square errors in estimating the parameters of the signal in Figure 7 is presented in Figure 10. The chirp rate and initial phase corresponding to the minimum point are the results of demand. Additionally, the estimated parameters $\hat{\gamma }=16.39\;\mathrm{rad}/s^{2}$, ${\hat{\varphi }}_{0}=4.95$ are quite close to the true value $\gamma =16.76\;\mathrm{rad}/s^{2},\;\varphi_{0}=4.94$. Actually, the estimated chirp rate can be also used in SISAR imaging. The traditional motion compensation method for SISAR based on contrast maximization [16] excessively relies on the imaging result, while it is not true for the proposed method of chirp rate estimation.

**Figure 11:**
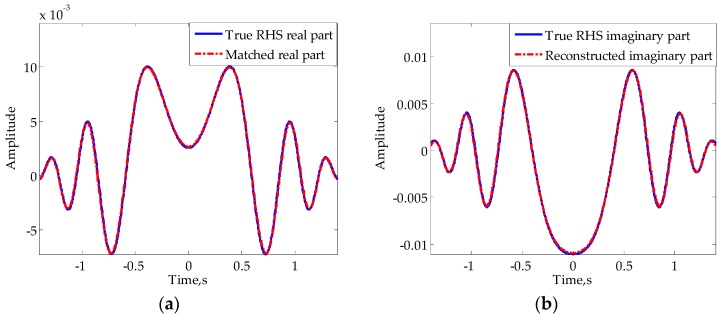
Results of (**a**) real part matching and (**b**) mainlobe RHS reconstruction. Solid lines represent true signals and dash-dotted lines represent constructed signals.

Figure 11 shows the results of real part matching and imaginary part reconstruction of the signal in Figure 7, which indicate that the matching signal of I(t) and reconstructed Q(t) in mainlobe both have high degrees of coincidence with the true values. Using the reconstructed imaginary part of RHS in mainlobe to substitute the distorted part after the segmented Hilbert transform, we can obtain the entire imaginary part of RHS.

### 3.3. Performance Analysis

According to the above analysis, to ensure the effectiveness and accuracy of the proposed method, it is quite necessary to make sure that the target is in the far-field. In [4], the Fresnel parameter was used to quantitatively analyze the scattering mechanism, which is expressed as:

(15) $$S=D^{2}/4\lambda$$

where *D* is the target’s largest effective size. The scattering mechanism was considered as a far-field diffraction for target distances larger than S. Similarly, the Fresnel number was used in [24] for the same purpose, written as:

(16) $$N_{F}=2w^{2}/\lambda L$$

where w is half of the target size in the direction perpendicular to the baseline. Using this definition, the scattering mechanism was considered as a far-field diffraction under the condition that $N_{F}<0.2$. It can be seen that neither definition has given full consideration to the target’s geometry relationship to the receiver and transmitter, thus being not accurate enough.

In order to provide a theoretical basis for the proposed RHS reconstruction method, we will next define a new parameter to differentiate between near-field scattering and far-field scattering in FSR and use it to give the scope of application for the novel RHS reconstruction method. 

In electromagnetic theory, when the incident wave is a plane wave, the scattering mechanism is considered as a far-field scattering if the secondary radiation wave of the target satisfies the plane-wave approximation. The target’s FS signal can be considered as the coherent superposition of signals scattered from numbers of silhouette elements of the target. Therefore, we can make use of the propagation path differences of signals from different silhouette elements to decide the scattering mechanism. From Equation (2) it can be seen that the FS signal can be approximately expressed by the integral of finite independent scattering sub-signals along the $x^{\prime }$ direction. Each sub-signal can be written as:

(17) $$E(t,x^{\prime })=Q\cdot H\left(x^{\prime }\right)\exp\left[j\frac{\gamma }{2}{\left(\frac{x^{\prime }}{v}+t\right)}^{2}\right]dx^{\prime }$$

Obviously, the propagation path difference of two sub-signals is:

(18) $$\Delta R\left({x^{\prime }}_{1},{x^{\prime }}_{2},t\right)=\frac{\gamma }{2k}\left[{\left(\frac{{x^{\prime }}_{1}}{v}+t\right)}^{2}-{\left(\frac{{x^{\prime }}_{2}}{v}+t\right)}^{2}\right]=\frac{\gamma }{2k}\left(\frac{{x^{\prime }}_{1}}{v}-\frac{{x^{\prime }}_{2}}{v}\right)\left(\frac{{x^{\prime }}_{1}}{v}+\frac{{x^{\prime }}_{2}}{v}+2t\right)$$

Giving ${x^{\prime }}_{1}=l/2$, ${x^{\prime }}_{2}=0$ and substituting them into Equation (18), we can obtain the propagation path difference of sub-signals from the center to the edge of the target after simplification:

(19) $$\Delta R\left(l/2,0,t\right)=\frac{\gamma l^{2}}{8kv^{2}}+\frac{\gamma l}{2kv}t$$

It can be found that, converting the distance to phase, the linear term of t on the right of Equation (19) has a periodic change as t increases. Therefore, only the case of t=0 is taken into consideration:

(20) $$\Delta R\left(l/2,0,0\right)=\frac{\gamma l^{2}}{8kv^{2}}$$

Based on the plane-wave assumption, we define a parameter $f_{L}$ as the ratio of ΔR(l/2,0,0) to a quarter of the wavelength, which is written as follows:

(21) $$f_{L}=\frac{\frac{\gamma l^{2}}{8kv^{2}}}{\lambda /4}=\frac{l^{2}\sin^{2}\phi }{2\lambda }\left(\frac{1}{d_{T}}+\frac{1}{d_{R}}\right)=\frac{l^{2}\sin^{2}\phi \cdot \Omega^{2}}{2\pi }$$

Compared to the last two definitions, its advantage is the consideration of the relative position of the target to the transceivers so that in certain situations it will be more precise. Apparently, an increasing $f_{L}$ indicates that the scattering mechanism is approaching near-field diffraction. Substituting the parameters in Table 1 into Equation (21), the corresponding $f_{L}$ of each signal in Figure 3 can be obtained as (a) 5.33, (b) 1.33, (c) 0.33, (d) 0.083. In general, we consider the target in the far-field for small $f_{L}$ (e.g., less than 0.1) and in the near field for large $f_{L}$ (e.g., greater than 1). To insure the effectiveness of our method, the parameter $f_{L}$ needs to be small enough. According to Equation (21), when the wavelength and baseline are fixed, $f_{L}$ is mainly determined by the target’s crossing position and trajectory angle ϕ. Obviously, we have large $f_{L}$ for ϕ close to 90° or a crossing point near the transmitter/receiver. In fact, the $f_{L}$ for ϕ=90° and $d_{T}=3d_{R}$ is only four thirds of the $f_{L}$ for ϕ=90° and $d_{T}=d_{R}$. That is to say, if the RHS for ϕ=90° and $d_{T}=d_{R}$ can be correctly reconstructed, there is a large possibility that the proposed method is effective for other situations. Therefore, for the sake of convenience, next we will still analyze the method’s performance under the situation of ϕ=90° and $d_{T}=d_{R}$.

For mainlobe RHS reconstruction, the necessary condition to guarantee its performance is that there should be enough samples of extremums in the mainlobe to acquire a fitting of high accuracy. We may assume that the minimum number of samples is four and that their corresponding times are $t_{1}<t_{2}<0<t_{3}<t_{4}$. We have the Doppler phase as:

(22) $$\varphi^{\prime }=\gamma t_{1}^{2}/2=\gamma t_{4}^{2}/2$$

According to the simple rule of quadratic phase change, we have $\varphi^{\prime }\in \left(\pi ,2\pi \right]$. According to Equation (5), the mainlobe duration of the first nulls can be approximately expressed as:

(23) $$T_{0}\approx 4\pi v/\left(\gamma l\right)$$

These extremums are in the mainlobe on condition that:

(24) $$t_{4}<T_{0}/2$$

Substituting $\gamma =2\Omega^{2}v^{2}\sin^{2}\phi$ and Equations (21)–(23) into Equation (24), we can obtain:

(25) $$f_{L}<\pi /2\varphi^{\prime }$$

Based on the restriction that $\varphi^{\prime }\in \left(\pi ,2\pi \right]$, the maximum of $f_{L}$ is 0.25, which means

(26) $$f_{L}=2l^{2}/\lambda L\leq 0.25$$

It can be seen that in this special situation Equation (26) degenerates to the simple definition in Equation (16). Since the new criterion is obtained by mathematical deduction, this equivalence also verifies the correctness of the traditional criterions from another perspective. However, for accurate analysis in a specific situation, Equation (21) should be used as a substitute. Additionally, it should be mentioned that, for the sake of simplicity, here we have used rectangle target and far-field approximation to obtain the approximate expression, Equation (23), for the calculation of mainlobe width. Therefore, the restriction given by Equation (26) should be used for rough judgment.

From another point of view, the parameter $f_{L}$ can be used for the design of FSR system parameters if the SISAR imaging is considered as one of the system functions. As concluded, the given method has better performance for smaller $f_{L}$, which means longer wavelength, longer baseline, and shorter target length. Therefore, once the configuration is fixed, we need a longer wavelength to achieve the correct RHS reconstruction and SISAR imaging for a longer targets. Considering an adverse situation in which the target is in the near field, the lack of extremums in the mainlobe will disable polynomial fitting and the estimation of the mainlobe envelope. Thus the performance of the proposed method will deteriorate sharply in this situation, and the same is true for the traditional method. Though a long wavelength can guarantee the method’s performance for a long target, the resolving effect of a short target becomes worse as the resolution is proportional to the wavelength [14]. To solve this dilemma, a multi-frequency system might be an option.

## 4. Simulation Results and Discussion

**Figure 12:**
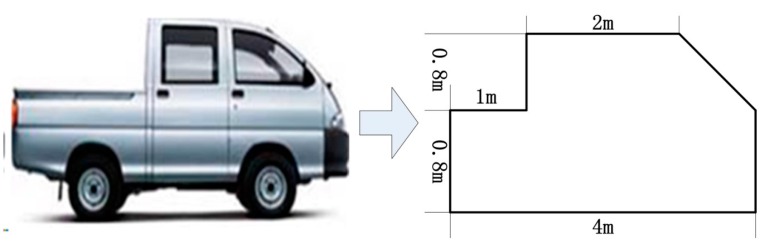
Simplified ground vehicle model.

As has been pointed out, the initial phase of RHS varies for different system parameters. To make a comprehensive comparison, we will simulate the RHSs with different initial phases by using different system parameters. In the simulation, the target is a ground vehicle as shown in Figure 12. The simulation parameters are listed in Table 2. Supposing the target crosses the midpoint of baseline perpendicularly with a velocity of 20 m/s, we have $f_{L}\approx 0.26$, which roughly meets the far-field diffraction condition given by Equation (26).

**Figure 13:**
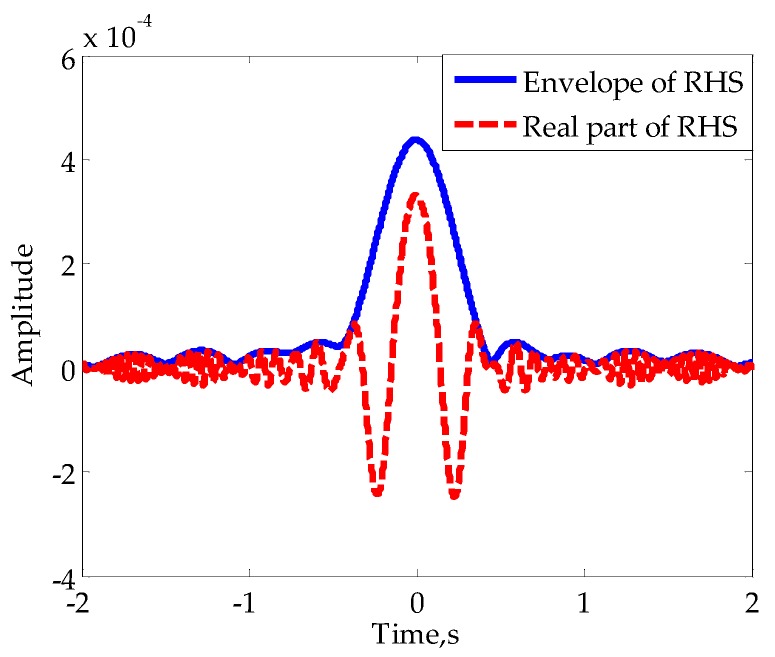
Simulated RHSs of vehicle model for baseline lengths of 400 m.

Substituting the above parameters into Equation (2) to simulate the target’s RHS, one of the real parts and envelopes is shown in Figure 13. To verify the performances of the proposed method for different initial phases of RHS, the baseline lengths were set to four values with a spacing of one-eighth of the wavelength. This means that the four initial phases sequentially differ π/4. Since the target is approximately in the far field, the RHS envelope is relatively smooth and there are at least four extremums in each mainlobe.

**Figure 14:**
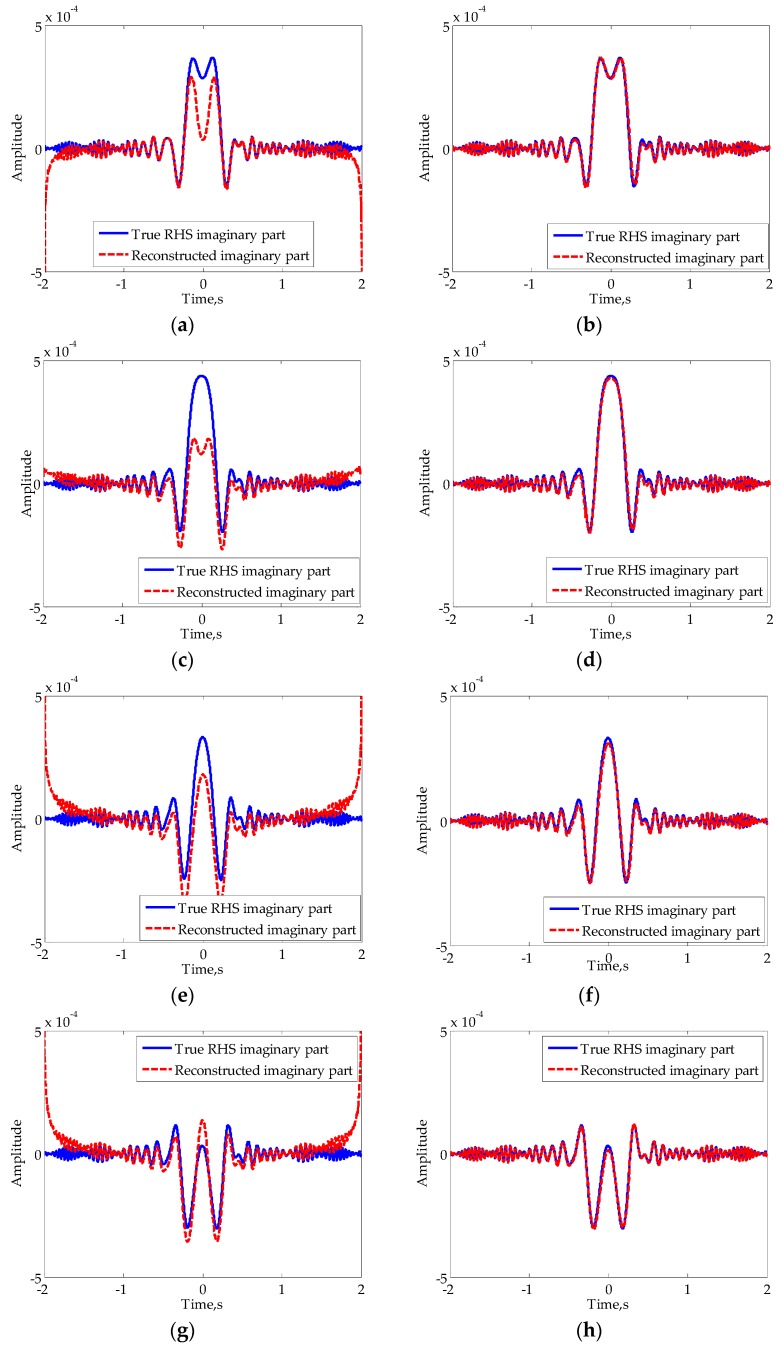
Reconstructed RHSs of vehicle model. In (**a**,**c**,**e**,**g**) the old method was used, while in (**b**,**d**,**f**,**h**) the new method was used. The baseline lengths are: (**a**,**b**) 400 m; (**c**,**d**) 400.0375 m; (**e**,**f**) 400.075 m; (**g**,**h**) 400.15 m.

For comparison, Figure 14 presents the reconstructed imaginary parts of RHS using the old method and the new method, respectively. As mentioned in [24], Approach 2 for the segmented Hilbert transform was applied. The order of polynomial fitting is five. The results indicate that each of the reconstructed RHS imaginary parts using the traditional method have obvious electric mean shifts, not only in the center of the signal, but also at both ends. Besides, since the initial phases of RHS are not zeroes, fitting using quadratic function cannot achieve accurate estimates of the imaginary parts of mainlobe RHS. In contrast, the reconstructed imaginary parts of RHS using the novel method fit the true values well, which validates the novel method’s advantage in RHS reconstruction.

As is known, the cross-correlation function can be used to analyze the degree of similarity of two signals. To mathematically compare the two techniques, we introduce the correlation coefficient ρ, which is defined by the normalized value of the cross-correlation function at zero time as follows [26],

(27) $$\rho =\frac{\int_{-\infty }^{+\infty }Q\left(t\right)\hat{Q}\left(t\right)dt}{\sqrt{\int_{-\infty }^{+\infty }Q^{2}\left(t\right)dt\cdot \int_{-\infty }^{+\infty }{\hat{Q}}^{2}\left(t\right)dt}}$$

where Q(t) is the true RHS quadrature component and $\hat{Q}\left(t\right)$ is the reconstructed RHS quadrature component. Obviously, a correlation coefficient that is more close to 1 represents a better RHS reconstruction performance. The correlation coefficients for the signals in Figure 14 are calculated and listed in Table 3. It can be seen that the correlation coefficients of the reconstructed signals by the new method are quite close to 1, indicating its good performance. However, the corresponding correlation coefficients for the old method are approximately three-quarters.

**Figure 15:**
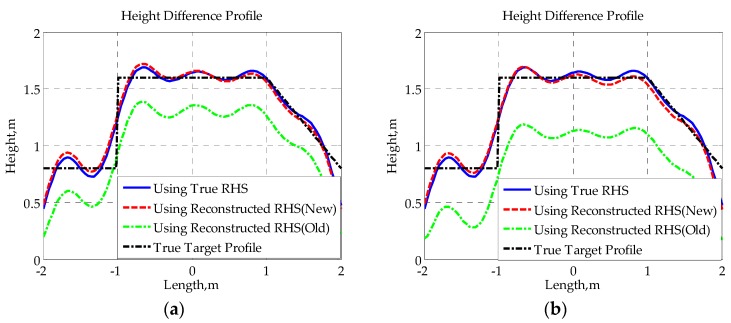

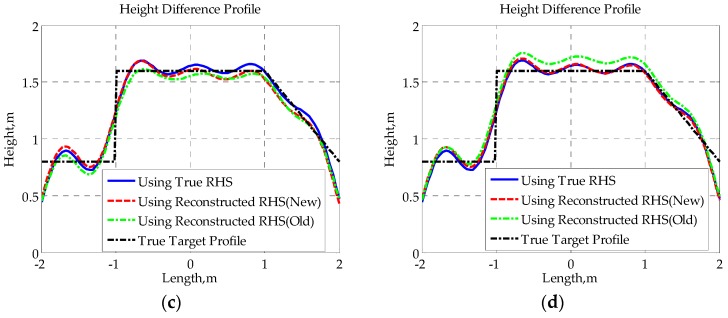
Height difference profiles for baseline lengths: (**a**) 400 m; (**b**) 400.0375 m; (**c**) 400.075 m; (**d**) 400.15 m.

**Figure 16:**
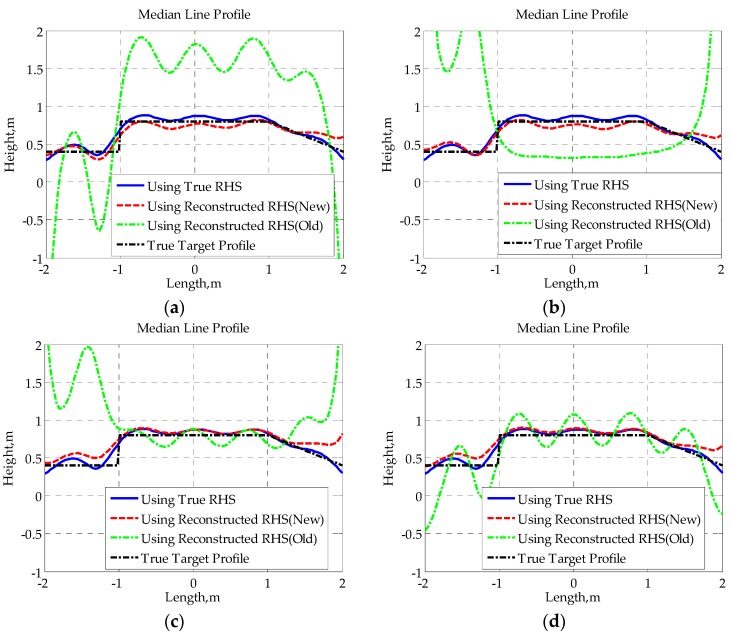
Median line profiles for baseline lengths: (**a**) 400 m; (**b**) 400.0375 m; (**c**) 400.075 m; (**d**) 400.15 m.

Subsequently, we use the complex RHSs, which are composed of the reconstructed imaginary part in Figure 14 and the true real part for SISAR imaging, and the corresponding results are given in Figure 15 and Figure 16. The true target profiles are represented by black dash-dotted lines. The restored profiles using true RHSs are represented by blue solid lines. The restored profiles using RHSs reconstructed by the new method and the old method are represented by red broken lines and green dash-dotted lines, respectively. Both the height difference profile and median line profile of each RHS are obtained. Supposing the upper edge and lower edge of the shadow profile are expressed by $h_{u}\left(x^{\prime }\right)$ and $h_{l}\left(x^{\prime }\right)$, the height difference profile $h_{d}\left(x^{\prime }\right)$ and median line profile $h_{m}\left(x^{\prime }\right)$ are defined as follows:

(28) $$h_{d}\left(x^{\prime }\right)=h_{u}\left(x^{\prime }\right)-h_{l}\left(x^{\prime }\right),\;h_{m}\left(x^{\prime }\right)=\left[h_{u}\left(x^{\prime }\right)+h_{l}\left(x^{\prime }\right)\right]/2$$

Obviously, once the height difference profile and median line profile are obtained, the target’s contour profile can be easily constructed using the following equation:

(29) $$h_{u}\left(x^{\prime }\right)=\left[2h_{m}\left(x^{\prime }\right)+h_{d}\left(x^{\prime }\right)\right]/2,\;h_{l}\left(x^{\prime }\right)\;=\;\left[2h_{m}\left(x^{\prime }\right)-h_{d}\left(x^{\prime }\right)\right]/2$$

From the given results, it can be found that there is not much difference between the shapes of height difference profiles obtained using different RHSs. Though the reconstruction of height difference profile is, to a certain extent, not sensitive to the RHS phase, those distortions in the reconstructed RHS using the traditional method may cause some loss of signal energy (as shown in Figure 15a,b), which will affect the extraction of the true target height. Furthermore, owing to the sensitivity of the median line profile to signal phase, the median line profile seriously deviates from the true profile when the traditional method is applied to reconstruct RHS. However, since the proposed method provides a much higher accuracy in RHS reconstruction, the median line profiles obtained with the novel method fit the true profiles well, verifying the effectiveness of the given method from another point of view. 

**Figure 17:**
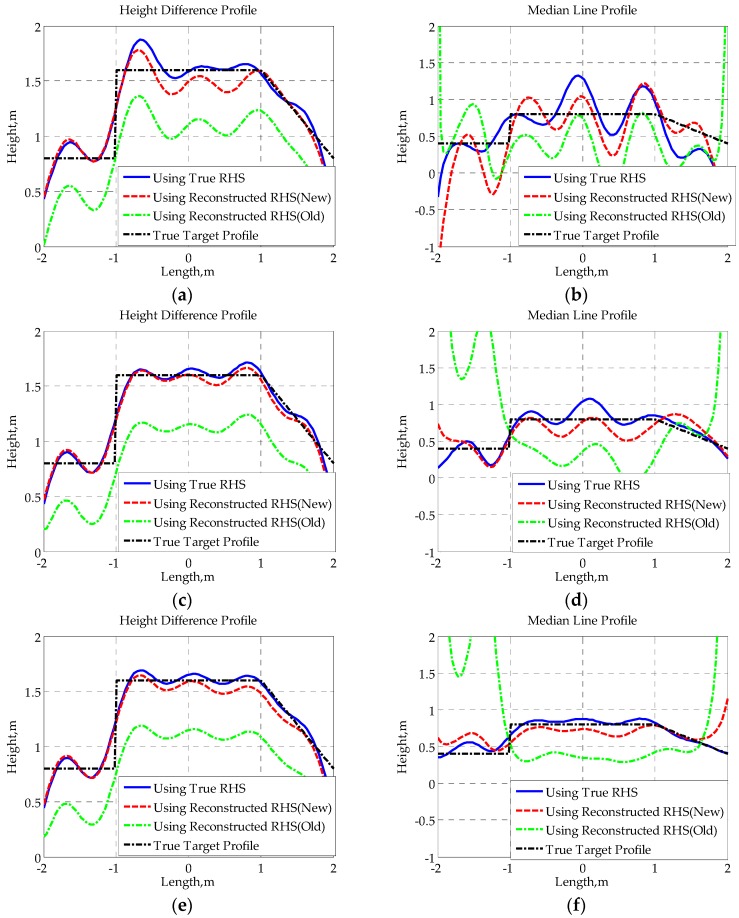
Shadow Inverse Synthetic Aperture Radar (SISAR) imaging results of signals with: (**a**,**b**) signal-to-noise ratio (SNR) = 0 dB; (**c**,**d**) SNR = 10 dB; (**e**,**f**) SNR = 20 dB. The height difference profiles are in the left column and the median line profiles are in the right column.

In practical application, the effect of noise should not be ignored. Actually, the mainlobe RHS reconstruction might not work normally, as the noise will interfere with the extraction of extremums. Therefore, in engineering applications, a low-pass filter should be used to smooth the received signal in mainlobe before the extraction of extremums. To further analyze the influence of noise, we simulated signals with specific signal to noise ratios (SNR) and used them for RHS reconstruction and SISAR imaging. The signal in Figure 14c was taken as an example. In RHS reconstruction, the bandwidth of the low-pass filter was set to 5 Hz according to the Doppler. Figure 17 shows the SISAR imaging results of signals with different SNRs.

It is shown that, as the imaging process can be considered as coherent integration, the extracted profiles have higher SNRs than the signals. Additionally, the results indicate that the height difference profiles are not quite sensitive to noise and are still acceptable at a 0 dB SNR. However, the median line profiles are much more easily affected by the existence of noise for the use of phases. A SNR of 10 dB can roughly guarantee the extraction of median line profiles. Besides, only minor differences are shown between the imaging results obtained using true RHSs and reconstructed RHSs of the new method; the same is true with the results in Figure 15 and Figure 16. This phenomenon indicates that the proposed method will not cause a deterioration of performance in case of noise.

## 5. Conclusions

In this paper, an improved method for RHS reconstruction is presented based on the analysis of signal characteristics. The specific sequence of the implementation of segmented Hilbert transform is adjusted and the mainlobe RHS is obtained with the estimation of signal envelope. Compared with the traditional method for RHS reconstruction, the novel method has a wider adaptability and a higher accuracy. Meanwhile, based on the analysis of performance, a method to distinguish between the near-field diffraction and far-field diffraction for FSR is given. Then, the applicable conditions for the method of RHS reconstruction are also specified. Verifications based on simulated data prove that the proposed method has obvious advantages over the traditional method on RHS reconstruction and SISAR imaging. To sum up, the proposed method provides an effective means of RHS reconstruction for FSRs with non-synchronized transceivers, making the implementation of SISAR imaging possible in more situations. Future works will concentrate on the collection of valid experimental data for the verification of the proposed method and SISAR imaging.

## Figures and Tables

**Table 1:** Simulation parameters of rectangular plates.

Parameter	Value	Parameter	Value	Parameter	Value	Parameter	Value
Wavelength (m)	0.2	Track angle (°)	90	Target center height (m)	0	Observation interval (s)	8
Baseline (m)	30	Velocity (m/s)	2	Rectangle height (m)	1	^1^ Center ratio	1:1

^1^ Center ratio means the ratio of transmit range to receive range at the crossing time.

**Table 2:** Simulation parameters of vehicle model.

Parameter	Value	Parameter	Value	Parameter	Value	Parameter	Value
Wavelength (m)	0.3	Track angle (°)	90	Target center height (m)	0.8	Observation interval (s)	4
Baseline (m)	~400	Velocity (m/s)	20	Target height (m)	1.6	Center ratio	1:1

**Table 3:** Parametric comparison of the two methods as displayed in Figure 14.

Figure	ρ	Figure	ρ	Figure	ρ	Figure	ρ
(a)	0.8310	(c)	0.7850	(e)	0.6861	(g)	0.8013
(b)	0.9976	(d)	0.9963	(f)	0.9939	(h)	0.9962

## Abbreviations

The following abbreviations are used in this manuscript:

FSR	Forward scatter radar
FS	Forward scattering
CPF	Contour profile function
SISAR	Shadow inverse synthetic aperture radar
RHS	Radio hologram signal
RCS	Radar cross section
